# Characterization of Thirty Germplasms of Millet Pepper (*Capsicum frutescens* L.) in Terms of Fruit Morphology, Capsaicinoids, and Nutritional Components

**DOI:** 10.3390/metabo15010047

**Published:** 2025-01-14

**Authors:** Ruihao Zhang, Mengjuan Li, Junheng Lv, Pingping Li, Yunrong Mo, Xiang Zhang, Hong Cheng, Qiaoling Deng, Min Gui, Minghua Deng

**Affiliations:** 1 Key Laboratory of Vegetable Biology of Yunnan Province, College of Horticulture and Landscape, Yunnan Agricultural University, Kunming 650201, China; zrh@yaas.org.cn (R.Z.); p202412@ynau.edu.cn (M.L.); junhenglv@ynau.edu.cn (J.L.); lpp@yaas.org.cn (P.L.); moyunrong@stu.ynau.edu.cn (Y.M.); xiang.zhang@stu.hunau.edu.cn (X.Z.); 2022210204@stu.ynau.edu.cn (H.C.); 2023210209@stu.ynau.edu.cn (Q.D.); 2 Horticulture Research Institute, Yunnan Academy of Agricultural Sciences, Kunming 650205, China

**Keywords:** millet pepper, morphological traits, nutritional components, genetic diversity, comprehensive evaluation

## Abstract

Background: Millet peppers have rich and diverse germplasm resources. It is of great significance to characterize their phenotypes and physicochemical indicators. Methods: 30 millet germplasms were selected to measure the fruit length and width, flesh thickness, number of ventricles, fruit stalk length, and single fruit weight, and the texture characteristics of fruit such as hardness, cohesiveness, springiness, gumminess, and chewiness were determined by a texture analyzer. At the same time, high-performance liquid chromatography (HPLC) and gas chromatography (GC) were used to determine the fruit of capsaicin, dihydrocapsaicin, nordihydrocapsaicin, fatty acids, vitamin E (VE), total phenol, total sugar, and total dietary fiber. Results: M11 showed outstanding parameters in phenotype and texture. The coefficient of variation (CV) for VE was as high as 94.943% and the highest diversity index (H’) was total soluble solid, at 1.988%. M5 and M18 contained rich and diverse fatty acids. At the same time, the content of capsaicinoids in M18 also ranks among the top, second only to M27 (with a total capsaicin content of 5623.96 μg/g). PCA analysis using phenotypic data and physicochemical data showed that the classification results were different. Further hierarchical group analysis was carried out using all the index data. The results showed that 30 millet pepper germplasms were divided into three new categories: M5, M9, M18, and M24 formed one group (C1), M10, M14, M16, M19, M20, M22, M25, M26, M28, M29, and M30 formed another cluster (C2), and the remaining germplasms formed a third cluster (C3). Among them, the abundance of fatty acids in the C1 germplasm was higher than that in the other two groups. Conclusions: Our study showed that different germplasms had significant differences in morphological traits and nutritional metabolic components and were rich in genetic diversity. This study provides a theoretical basis for the improvement of millet varieties and the development of functional food.

## 1. Introduction

Millet pepper (*Capsicum frutescens* L.), also known as “Xiaomila”, is an annual or perennial shrubby pepper of the genus Capsicum in the Solanaceae, which is mainly distributed in tropical areas, such as Yunnan and Hainan Provinces in China [1]. The flower and fruit of millet pepper are erect or lateral, and the fruit is short finger-shaped, short cone-shaped, or rice grain-shaped [2]. As one of the five domesticated species of Capsicum (which are *C. annuum*, *C. baccatum*, *C. chinense*, *C. frutescens*, and *C. pubescen*) [3], millet pepper is an important vegetable and spice crop, which is widely popular in the world. It is not only used as a condiment to add flavor to dishes but also famous for its unique spicy flavor and rich bioactive ingredients [4]. Capsaicinoids, vitamin C, vitamin E, phenolic compounds, dietary fiber, and other bioactive components are rich in millet pepper [5]; these ingredients together provide various health benefits for the human body, such as antioxidant, anti-inflammatory, and anti-cancer benefits. In addition, *C. frutescens* is used as an external treatment for muscle pain in some areas [6]. It is also used to treat hyperglycemia, high/low blood pressure, bronchitis, and burns, as well as to promote blood circulation, relieve rheumatic discomfort, etc. [7]. Many studies have shown that chili peppers possess high nutritional value, health benefits, and medicinal properties [3,6,8], which have established a niche for millet pepper in the field of healthy foods. With the rapid development of the pepper processing industry, the quality requirements for millet pepper have become increasingly stringent. Therefore, in-depth research on their quality characteristics and bioactive components is of great significance for breeding superior varieties, enhancing their nutritional value, and developing healthy food products.

In recent years, research on the quality components of millet peppers has mainly focused on the analysis of active components such as capsaicinoids, vitamins, amino acids, polyphenols, and minerals. For instance, Liu et al. [9] conducted an analysis of genetic diversity and quality components of millet pepper resources in Yunnan, revealing differences in genetic background and quality components among millet peppers from different geographical origins. Wu et al. [10] studied the synthesis patterns of capsaicinoid compounds in millet peppers. Karaman et al. [11] investigated the phenolic and tocopherol compositions, capsaicinoid profiles, and bioactive components of recombinant inbred lines of *C. annuum* and *C. frutescens.* During the processing of millet peppers, textural properties are one of the important factors affecting consumer acceptance. Textural indicators such as hardness, elasticity, and chewiness directly influence the taste and quality of the product. Bai [12] studied the impact of fermentation conditions on the textural properties of Yunnan pickled millet peppers, providing reference information for the quality control of fermented chili products. Ye et al. [13] analyzed the effects of different varieties and ripeness levels on the texture, physicochemical properties, and flavor of pickled millet peppers.

Despite these efforts, systematic research on the phenotypic traits, textural properties, and intrinsic quality components of millet pepper germplasm resources remains insufficient. These traits are not only influenced by various factors such as variety, environmental conditions of origin, and cultivation practices, but also their genetic diversity determines the richness and stability of these traits. To further explore the impact of genetic diversity on the appearance and intrinsic quality of millet peppers, this study selected 30 representative millet pepper germplasms from Yunnan and comprehensively measured and analyzed key indicators such as fruit length, width, flesh thickness, number of ventricles, fruit stalk length, and single fruit weight. These traits are crucial for assessing the yield potential and commercial value of varieties. Additionally, texture analysis technology was employed to determine the hardness, elasticity, chewiness, adhesiveness, and cohesiveness of the fruits, evaluating the differences in taste and processing characteristics among different germplasms. To fully assess the nutritional value of millet peppers, this study also quantitatively analyzed the pungent components such as capsaicin, dihydrocapsaicin, and nordihydrocapsaicin in the fruits. The content of bioactive components such as fatty acids, vitamin E, total phenols, total sugars, and total dietary fiber in the fruits was also determined. By comprehensively evaluating these traits and quality components, the aim is to reveal differences among various millet pepper germplasms, providing a scientific basis for variety improvement. Moreover, the research findings will also provide important references for the development of functional foods, offering new perspectives for food processing and the development of healthy food products.

## 2. Materials and Methods

### 2.1. Plant Material

**Figure 1:**
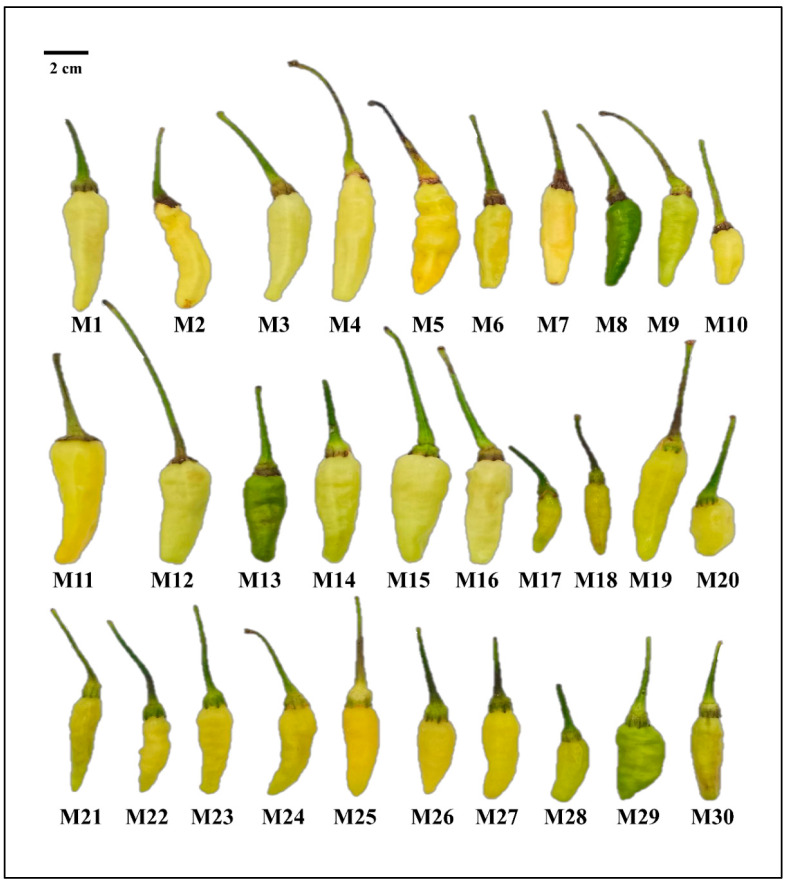
Photographs of the millet pepper germplasms included in this study.

The 30 millet pepper germplasm resources used in this study (Figure 1) were provided by the pepper research group at the Horticulture Research Institute, Yunnan Academy of Agricultural Sciences. M1–M30 are the abbreviations for the 30 millet pepper germplasms. The experimental materials were sown and cultivated in Yuanjiang County, sown on 1 October 2022, transplanted on 14 November, and harvested on 10 April 2023. The planting method was to open deep furrows and build narrow ridges, plant double rows on the ridge, and plant with distances of 50 cm × 60 cm and 20 plants per box planting. The same cultivation conditions and water and fertilizer management measures were adopted for all tested peppers. The fruits at green maturity (40 days after flowering) were harvested as the experimental samples. Five healthy plants were selected from each variety, and 10 fruits were harvested from each plant. The fruits were required to be similar in maturity and uniform in size. The samples were stored at −80 °C for subsequent experiments.

### 2.2. Morphological Index Determination

The fruit length, fruit width, flesh thickness, number of ventricles, and individual fruit weight were assessed according to the previously described method [14]. Fruit stalk length was measured from the calyx to the plant stem using a flexible ruler. Ten biological replicates were evaluated for the aforementioned parameters.

### 2.3. Texture Profile Analysis (TPA)

The hardness, cohesiveness, springiness, gumminess, and chewiness of the pepper fruits were measured using a texture analyzer fitted with a spherical probe (TMS pro, FTC, Sterling, VA, USA). Each pepper was measured at the equatorial part of the fruit (the place with the largest transverse diameter). A two-cycle compression test was conducted according to the method reported [15,16], with some modifications. The test conditions were as follows: standard disc probe; pretest speed, test speed, and post-test speed were 5 mm/s, 1 mm/s and 5 mm/s, respectively; the strain compression was 15%; the pause time of two compressions was 2 s; and the trigger force was 1 N.

### 2.4. Nutritional Quality Indicator Determination

The chlorophyll and carotenoid contents were measured according to the method of Zhong et al. [17]. Total soluble solids (TSSs), titratable acid, total sugar, and total phenols were determined according to the previously described method [18]. Vitamin E was measured using Vitamin E assay kits (catalog no.: A008-1-1, www.njjcbio.com). Total dietary fiber (TDF) content was determined and calculated by the enzymatic–gravimetric method following the GB 5009.88-2023 [19].

### 2.5. Fatty Acid Determination

Fatty acid components were identified and quantified following the GB 5009.168-2016 [20] National Food Safety Standard (second method), using a THERMO TRACE 1600 (Thermo Fisher Scientific, Waltham, MA, USA) gas chromatograph equipped with a flame ionization detector (FID, Trace GC 2000, Thermo Finnigan, Bremen, Germany). The samples were saponified and methyl esterified in a 2% potassium hydroxide methanol solution to produce fatty acid methyl esters, which were analyzed by TR-FAME GC column (100 m × 0.25 mm × 0.20 μm, Thermo) gas chromatography and quantified by the external standard method to determine the percent fatty acid content. The operating conditions were as follows: carrier gas high-purity nitrogen flow rate of 1.0 mL/min; initial temperature of 60 °C for 5 min; increase to 160 °C at a rate of 10 °C/min, hold for 2 min; increase to 200 °C at a rate of 2 °C/min, hold for 15 min; injection temperature 260 °C; detector temperature 280 °C.

### 2.6. Capsaicinoids Determination

Capsaicin, dihydrocapsaicin, and nordihydrocapsaicin contents were evaluated using the HPLC system as detailed previously according to the method described by Li et al. [18]. Capsaicinoid compounds were identified according to the retention times of the standards and quantified according to the standard curve. Each sample was conducted with three biological replicates. The capsaicinoids were expressed as μg/g.

### 2.7. Statistical Analysis

The morphological indices and capsaicinoids were analyzed and plotted by GraphPad Prism 9.0 software. The data of TPA were processed by IBM SPSS 26 and statistical evaluation of the results was conducted by using Tukey’s test (*α* = 0.05). Microsoft Excel 2019 was used for data analysis, and the minimum (Min), maximum (Max), mean values (Mean), standard deviation (SD), standard error (SE), coefficient of variation (CV), and the Shannon diversity index (H’) were calculated. The Shannon diversity index was calculated according to the method described by Jin et al. [21]. The construction of the principal component analysis (PCA) model was performed using the SIMCA software (Umetrics, Sartorius Stedim Biotech AS, Umea, Sweden) version 14.1. Heatmap and hierarchical group analysis on all measured indicators and samples were performed based on the R language version 4.1.0 and online software ImageGP 2.0 with Spearman correlation coefficients as a distance measure [22].

## 3. Results

### 3.1. Fruit Morphological Traits Measurement

**Figure 2:**
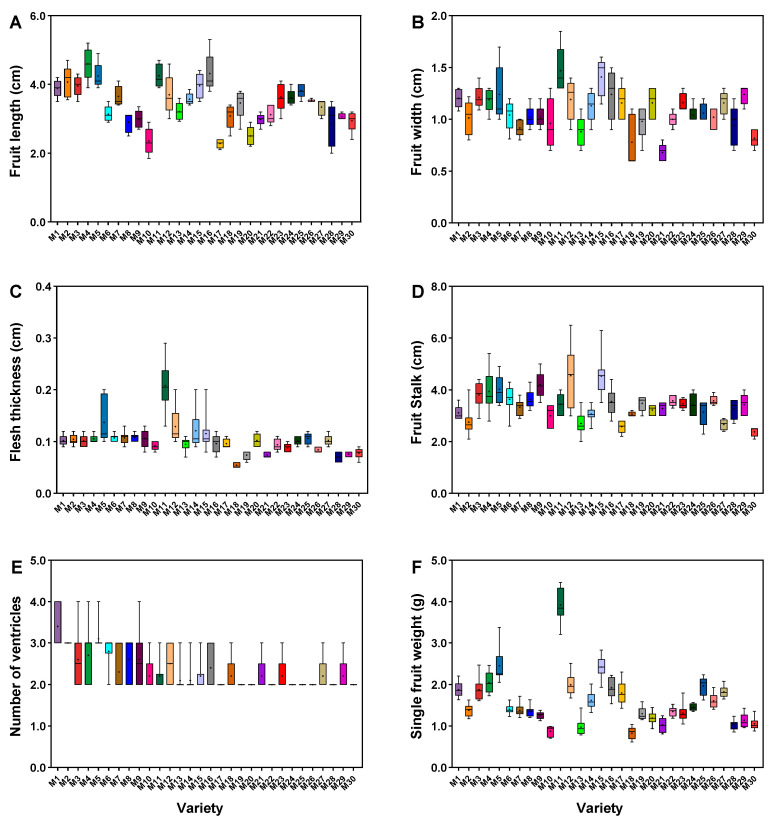
Morphological indicator measurements of fruit for the 30 millet pepper germplasms. Fruit length (**A**), fruit width (**B**), fruit flesh thickness (**C**), fruit stalk (**D**), number of ventricles (**E**), single fruit weight (**F**). The black dots represent the average values.

The fruit length, fruit width, flesh thickness, stalk length, number of ventricles, and single fruit weight of 30 millet pepper germplasms were measured in this study (Table S1). As shown in Figure 2, among the germplasms, M4 (4.59 cm) had the longest fruit length, followed by M16 (4.30 cm) and M11 (4.25 cm), and M17 (2.28 cm) had the shortest fruit length. The maximum fruit width was 1.47 cm for M11, and the minimum was 0.68 cm for M21; the maximum flesh thickness was 0.21 cm for M11, 0.14 cm for M5, and 0.05 cm for M18; the length of fruit stalk ranged from 2.38 cm to 4.55 cm, where M12 was the longest and M30 was the shortest; the number of ventricles in all the germplasms was more than two, of which having more than three were M1, M2, and M5, and the rest had between two and three; the highest single fruit weight was M11 (3.92 g), followed by M5 (2.45 g), M15 (2.42 g), and M18 (0.83 g).

### 3.2. Texture Properties

Millet peppers are renowned for their crisp and refreshing texture, which was an important evaluation criterion whether consumed fresh or processed into pickled pepper products. Therefore, we used a texture analyzer to measure the hardness, cohesiveness, springiness, gumminess, and chewiness of 30 millet germplasms. As shown in Table 1, the range of hardness was between 4.52 and 18.49 N, with M11 having the highest hardness, significantly different from the hardness of the other peppers. Next were M5 (12.45 N) and M3 (11.88 N), while M17 had the lowest hardness. The highest cohesiveness was found in M2 and M27 with 0.76, and the weakest was found in M7 and M9 with 0.53; the overall difference among the 30 peppers was not significant. M11 had the highest springiness at 1.62 mm, followed by M15 (1.48 mm) and M12 (1.39 mm), with no significant differences between them, while M18 had the lowest springiness at only 0.56 mm. The highest gumminess was recorded for M11 at 11.28 N, significantly higher than the other peppers and the lowest gumminess was for M17 at 2.94 N. There was a large variation in chewiness among the 30 peppers, with the highest being M11 at 18.16 mj, significantly higher than the others, and the lowest chewiness was for M18 at 1.89 mj.

### 3.3. Diversity Analysis of Nutritional Indicators

This study determined and analyzed nutritional indicators such as chlorophyll a, chlorophyll b, total carotenoids, soluble solids, titratable acidity, total sugars, total phenols, vitamin E, and total dietary fiber in millets (for details, see Table S2). As shown in Table 2, the content of chlorophyll a ranged from 0.068 to 0.546 mg/L, while the content of chlorophyll b varied from 0.035 to 0.408 mg/L, and the total carotenoid content was between 0.007 mg/L and 0.377 mg/L. The content ranges of soluble solids, titratable acidity, total sugars, total phenols, and total dietary fiber were relatively small, being 3.100–8.730%, 0.268–0.804%, 6.008–11.900 mg/g, 1.363–2.905 mg/g, and 5.400–15.140 g/100 g, respectively. The content of vitamin E showed the greatest variation, with the highest value reaching 217.334 μg/g and the lowest value only 13.965 μg/g. The coefficient of variation was the highest for vitamin E at 94.943% and the lowest for total dietary fiber at 21.459%. The diversity index (H’) was the greatest for total soluble solids at 1.988 and the smallest for vitamin E at 1.345.

### 3.4. Fatty Acid Content

In this study, the fatty acids in the fruits of 30 millet germplasms were determined, and a total of 22 fatty acids were detected, with specific contents shown in Table 3. Overall, butyric acid (C4:0), hexanoic acid (C6:0), palmitic acid (C16:0), and α-linolenic acid (C18:3n3) are the common fatty acids found in all peppers. Specifically, the highest content of C4:0 was 42.12 μg/g in M9, and the lowest was 5.08 μg/g in M27; the highest content of C6:0 was 24.33 μg/g in M9, and the lowest was 3.33 μg/g in M27. There were significant differences in the content of palmitic acid (C16:0) and α-linolenic acid (C18:3n3) among the tested millets. The content of C16:0 ranged from 0.16 μg/g to 798.46 μg/g, with the highest content found in M5 and the lowest in M29. The content of C18:3n3 was highest in M18 at 253.01 μg/g, followed by M5 at 194.07 μg/g and M9 at 166.67 μg/g, with the lowest content in M16 at 0.93 μg/g. Additionally, except for M16 and M17, all other samples contained arachidonic acid (C20:4n6), with the highest content being 32.42 μg/g in M21 and the lowest at 0.70 μg/g in M11. In contrast, capric acid (C10:0) was only found in small amounts in M5, M9, and M18, with contents of 2.93 μg/g, 2.94 μg/g, and 0.69 μg/g, respectively. The distribution of the remaining fatty acids also varied among different germplasms, and M5 and M9 contained almost all of the detected 22 fatty acids, except for trans-linoleic acid (C18:2n6t).

The saturated fatty acids (SFAs) detected in the samples include 12 types of fatty acids. There was a significant difference in the content of SFAs among the 30 millet germplasms, with M5 having the highest content at 1097.17 μg/g and M25 having the lowest content at only 10.66 μg/g. The monounsaturated fatty acids (MUFAs) detected include five types, with the highest content being 571.27 μg/g in M5, followed by 261.68 μg/g in M9 and 182.16 μg/g in M18. In contrast, M10, M14, M16, M19, M20, M22, M25, M26, M28, M29, and M30 did not contain any monounsaturated fatty acids. The polyunsaturated fatty acids (PUFAs) also consisted of five types of fatty acids, and there was a significant difference in their content among different germplasm resources, ranging from 0.93 μg/g to 2751.91 μg/g. The top-ranking germplasms in terms of PUFA content were M5, M18, M9, and M24.

### 3.5. Capsaicinoid Contents

**Figure 3:**
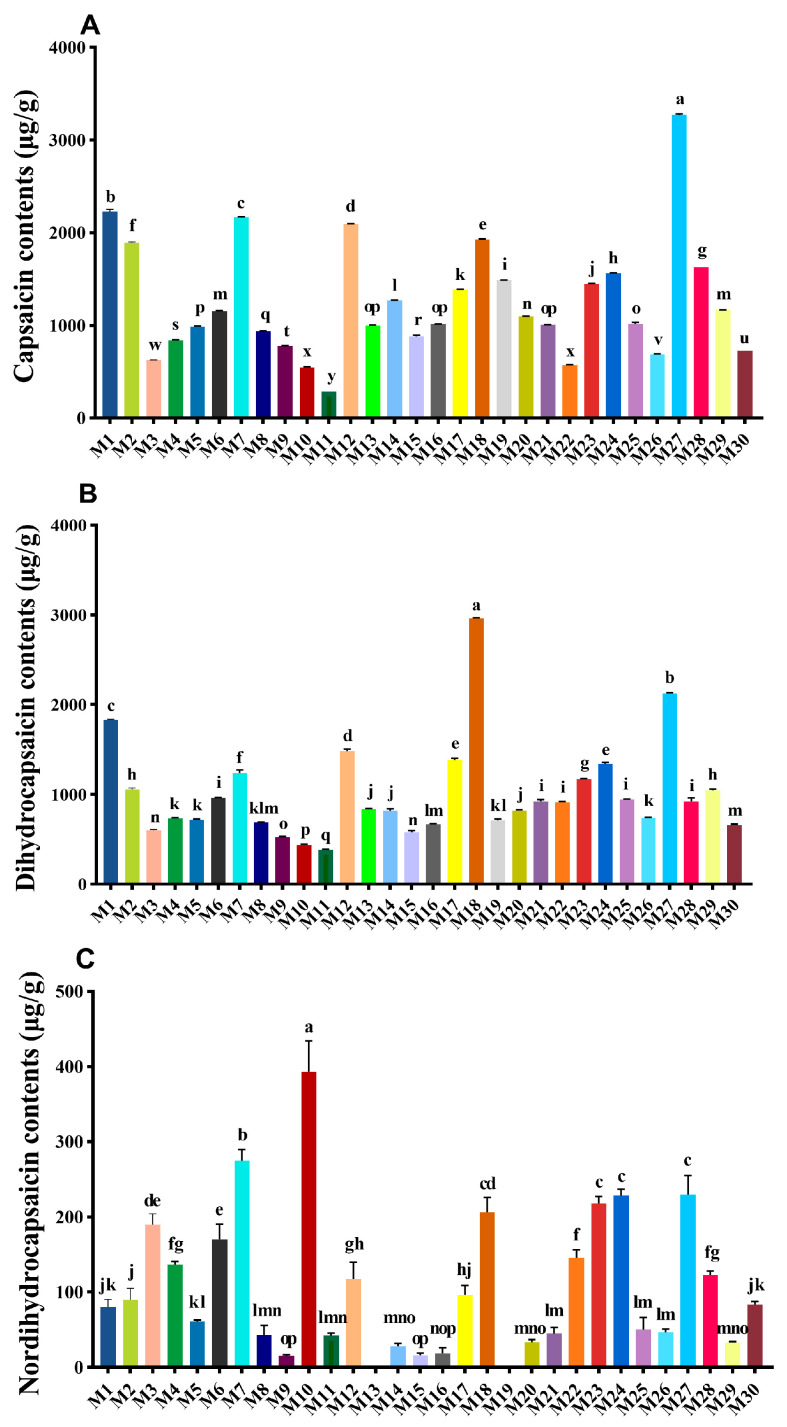
Capsaicin contents (**A**), dihydrocapsaicin contents (**B**), and nordihydrocapsaicin contents (**C**) in the 30 pepper germplasms. Data are expressed as average values (n = 3). Standard deviations are indicated by bars. Different lowercase letters indicate significant differences among germplasms (*p* < 0.05).

The total capsaicin contents and the contents of capsaicinoid monomers (including capsaicin, dihydrocapsaicin, and nordihydrocapsaicin) in 30 millet germplasms are shown in Figure 3. The total capsaicin content in M1, M7, M12, M18, and M27 was significantly higher than in other samples. Among them, M27 had the highest total capsaicin content, reaching 5623.96 μg/g, followed by M18 with 5094.96 μg/g and M1 with 4140.53 μg/g (Table S3). The lowest total capsaicin content was found in M11, with 706.67 μg/g, and its capsaicin and dihydrocapsaicin content was also the lowest among all samples. The content of capsaicin ranged from 284.13 μg/g to 3271.86 μg/g, with the highest content in M27, followed by M1 with 2228.94 μg/g and M7 with 2164.6 μg/g. The top three for dihydrocapsaicin content were M18 (2960.74 μg/g), M27 (2122.91 μg/g), and M1 (1831.54 μg/g). The highest content of nordihydrocapsaicin was found in M10 with 393.17 μg/g, followed by M7 and M27 with 274.94 μg/g and 229.19 μg/g, respectively. M13 and M19 did not detect nordihydrocapsaicin.

### 3.6. Principal Component Analysis (PCA)

**Figure 4:**
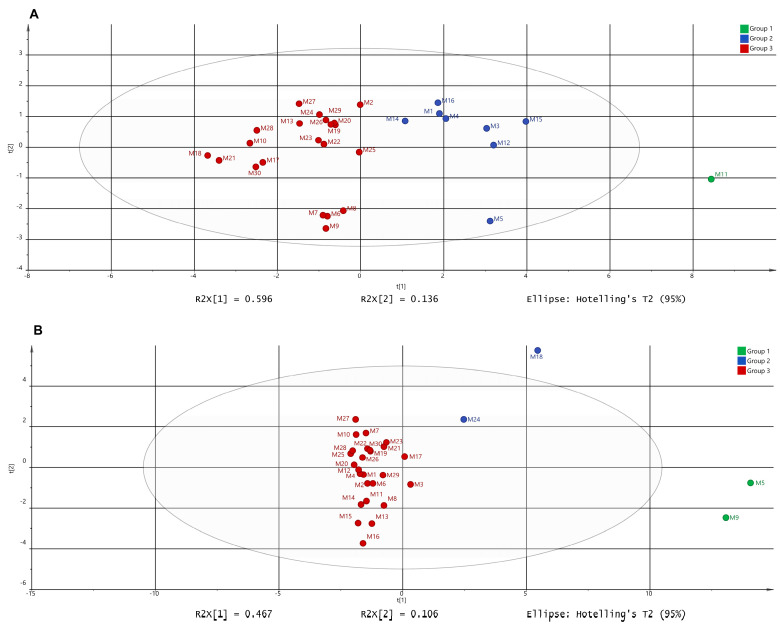
PCA of the appearance morphology and texture analyzer data (**A**), nutritional quality indicators, capsaicinoids, and fatty acids (**B**) of the 30 pepper germplasms analyzed.

We conducted a PCA on the morphological traits (fruit length, fruit width, flesh thickness, stalk length, number of ventricles, and single fruit weight) and texture parameters (hardness, cohesiveness, springiness, gumminess, and chewiness) of the fruits from 30 millet germplasms, respectively. The results, as shown in Figure 4A, indicated that the first principal component accounted for 0.596 of the variance, and the second principal component accounted for 0.136, with both together explaining 0.732 of the variance. Based on Ward’s method, the 30 germplasms were divided into three groups: M11 formed a single group; M1, M3, M4, M5, M12, M14, M15, and M16 formed another group; the remaining germplasm resources formed a third group. Additionally, we performed PCA on nutritional indicators, fatty acids, and capsaicinoid substances. The results, as shown in Figure 4B, indicated that the first principal component accounted for 0.467 of the variance, and the second principal component accounted for 0.106, with both together explaining 0.573 of the variance. The results still grouped the 30 germplasms into three categories, with M5 and M9 forming one group, M18 and M24 forming another group, and the remaining germplasms forming a third group.

### 3.7. Heatmap Analysis Between All Measured Indicators and 30 Millet Germplasms

**Figure 5:**
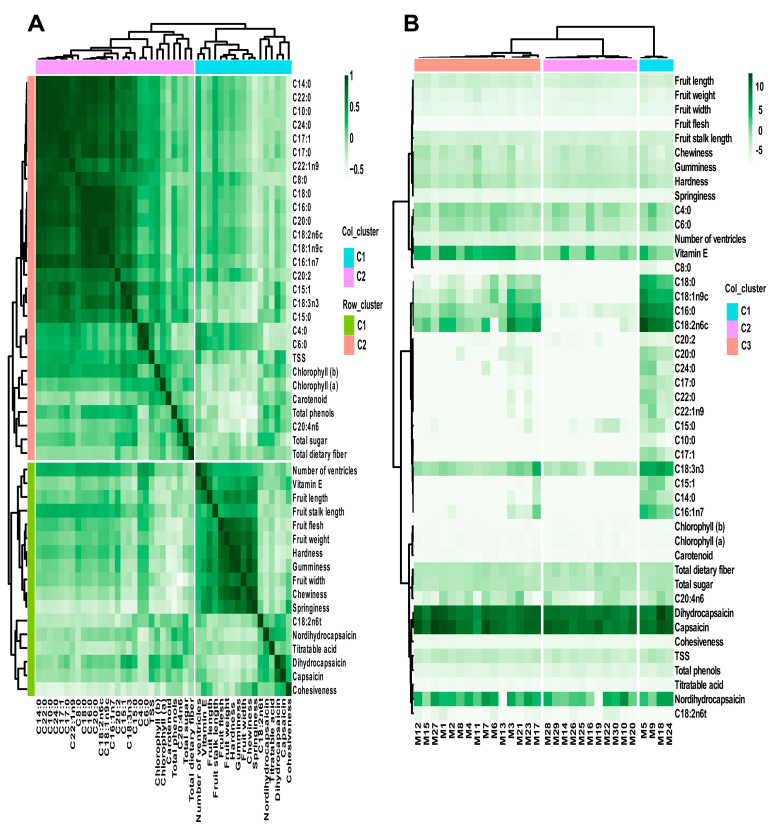
Correlation heatmap (**A**) and hierarchical cluster analysis (**B**) between appearance morphology and metabolite contents of the 30 millet pepper germplasms.

To explore the interrelationships among various indicators, a correlation heatmap analysis was conducted on all measured indicators of the 30 millet germplasms, with the results shown in Figure 5A. Except for C20:4n6 and C18:2n6t, the other fatty acids were positively correlated with each other, and these fatty acids were also positively correlated with soluble solids and chlorophyll a. In contrast, C20:4n6 and C18:2n6t were positively correlated with total sugars, capsaicin, dihydrocapsaicin, and nordihydrocapsaicin. Total phenols, total sugars, total dietary fiber, and titratable acidity were positively correlated with each other, and total phenols were also positively correlated with dihydrocapsaicin and nordihydrocapsaicin. Total dietary fiber was not only positively correlated with the aforementioned three metabolites but also with hardness and chewiness. Vitamin E is positively correlated with some fatty acids, with a stronger correlation with C4:0 and C6:0. Moreover, vitamin E is positively correlated with all phenotypic and textural indicators, with the strongest correlation with the number of ventricles (Table S4). Regarding phenotypic and textural indicators, all indicators were positively correlated with each other except for cohesiveness. Among them, flesh thickness and single fruit weight were strongly correlated with hardness, elasticity, adhesiveness, and chewiness.

Concurrently, hierarchical cluster analysis was performed using all morphological, textural, and metabolite indicators of the 30 millet germplasms, with the results shown in Figure 5B. The germplasms were broadly divided into three clusters: M5, M9, M18, and M24 formed one group (C1); M10, M14, M16, M19, M20, M22, M25, M26, M28, M29, and M30 formed another cluster (C2); and the remaining germplasms formed a third cluster (C3). This classification result differs from the PCA results. The clustering heatmap indicates that the C1 cluster of millet germplasms had higher contents of fatty acids such as C15:1, C14:0, C16:1n7, C17:1, C18:3n3, C22:0, and C22:1n9 compared to the C2 and C3 clusters. The C2 cluster of millet germplasms had lower contents of C18:0, C18:1n9c, C16:0, and C18:2n6c compared to the C1 and C3 clusters. The C3 cluster of germplasms overall had a higher content of vitamin E compared to the other two clusters. Additionally, capsaicin and dihydrocapsaicin were present in high amounts across all three groups of chili germplasms.

## 4. Discussion

The size of the pepper fruit is an important commercial trait for its appearance and also a significant consideration for its use as a raw material in processed products. Therefore, breeding varieties with sizes that meet market demands is one of the objectives in the development of new millet pepper varieties. The rich diversity in size of the millet pepper germplasms in this study provides a foundational condition for breeding new varieties of varying sizes. The flesh thickness of millet fruits is typically around 0.1 cm, while M11 has a flesh thickness exceeding 0.2 cm, which could be considered for preservation as a special resource for breeding varieties that require thicker flesh. It has been reported that pedicel length is one of the important indicators for germplasm identification, with moderate heritability [23]. In this study, the pedicel length of the germplasm materials also showed a significant range of variation, with considerable differences between different materials. Overall, the 30 pepper germplasms exhibit significant differences in fruit length, flesh thickness, stalk length, and single fruit weight, while the variation in the number of ventricles is relatively smaller, which is consistent with previous research findings [14,24]. The hardness of the fruit can reflect its freshness, and hardness is crucial for fruit quality [25]. M11 has the strongest hardness, elasticity, adhesiveness, and chewiness, demonstrating its potential in terms of storage and processing resistance. Under the same cultivation measures and habitats, the differences in phenotype and texture of these pepper materials indicate that different genotypes determine the expression of these traits.

The coefficient of variation (CV) is a critical measure that enables the direct comparison of the variability across different traits. In this study, the CV for various traits was exceedingly high (ranging from 21.459% to 94.943%), which is generally unexpected for such biochemical traits, as they are typically regulated by genetic factors. Among them, the CV values for total sugar, total dietary fiber, total phenols, and total soluble solids (TSS) were relatively low, indicating that the variation in these traits among the 30 millet pepper germplasms was minimal. In contrast, the CV for vitamin E was notably high, suggesting a highly uneven distribution of vitamin E abundance, with significant differences among the germplasms. These results precisely demonstrated the rich and diverse genetic background of the millet pepper. On the other hand, it may also be related to the adaptive strategies of millet peppers to the geographical environment and their resource utilization capabilities, which are the result of long-term adaptation to their living environment [26]. Therefore, traits with a higher CV value will provide valuable information for the identification and evaluation of germplasms. The Shannon–Weaver diversity index (H’) is widely used to assess the diversity of plant phenotypic traits and metabolites and is one of the most commonly applied methods for analyzing germplasm diversity [21]. In this study, the H’ for vitamin E was low, indicating low diversity of this trait among the millet pepper germplasms, as germplasms with either high or low vitamin E content accounted for a relatively large proportion (Table S2). Interestingly, while the CV value for vitamin E was very high, the H’ value was low. This phenomenon is reasonable within the millet pepper germplasm population. Given the significant habitat variation across the distribution range of millet pepper in Yunnan Province [9], combined with the effects of human selection, it is likely that germplasms with extremely high or low levels of metabolites dominate, while those with intermediate metabolite levels are either rare or absent. This reflects the specific ecological status of millet pepper germplasm, which warrants further ecological analysis to explore the underlying causes.

Studies have shown that foods rich in unsaturated fatty acids can reduce the risk of cardiovascular diseases [27] and may even help prevent the risk of SARS-CoV-2 infection [28]. The fruits of M5 and M9 contain 21 types of fatty acids, with both the content and diversity of unsaturated fatty acids being the most outstanding among the 30 millet pepper germplasms analyzed in this study. We propose that these two germplasms should be considered ideal breeding materials and valuable raw ingredients for the development of health-focused food products.

The capsaicinoid content in peppers is one of the key factors determining their commercial quality, and capsaicinoids are also the main active compounds responsible for their pharmacological properties [29]. The content of capsaicinoids primarily depends on the concentrations of capsaicin, dihydrocapsaicin, and nordihydrocapsaicin, which together account for approximately 97.5% of the total capsaicinoid content [30]. Their accumulation levels are largely influenced by genotype and various environmental factors, including light, soil moisture, and temperature [31,32]. Jeeatid et al. [33] reported that genotype significantly affects the capsaicin content in peppers. Similarly, Ye et al. [13] demonstrated that the cultivar plays a significant role in determining the pungency of millet pepper. In this study, the total capsaicinoid content among the 30 millet pepper germplasms ranged from 706.67 μg/g to 5623.96 μg/g, indicating substantial variation and highlighting the significant influence of genotype on capsaicinoid levels in these germplasms. The levels of capsaicin and dihydrocapsaicin were consistently higher than those of nordihydrocapsaicin, and in most samples, the capsaicin content exceeded that of dihydrocapsaicin. However, M18 was an exception, with a capsaicin content of 1928.12 μg/g and a remarkably high dihydrocapsaicin content of 2960.74 μg/g, nearly twice the capsaicin level. As a primary active component in pepper, dihydrocapsaicin exhibits various pharmacological and physiological effects, such as antioxidant activity [34,35]. Therefore, M18 holds potential advantages for the development of functional food products.

The PCA of external traits such as phenotype and texture (Figure 4A), compared with the PCA of internal traits such as metabolites (Figure 4B), divided the 30 millet pepper germplasms into three distinctly different groups. This indicates that the traits of these germplasms exhibit significant differences, reflecting their rich genetic diversity. Analyzing these germplasms based solely on external traits or metabolites would lead to biased conclusions. Therefore, a comprehensive analysis combining both external and internal traits is essential to achieve a relatively objective evaluation. To this end, we further performed hierarchical clustering analysis using all measured traits, resulting in a new classification of the 30 millet pepper germplasms into three groups (Figure 5B).

To better understand the interactions among the measured traits, we performed a correlation heatmap analysis. The plant cell wall is a complex structure composed of cellulose, hemicellulose, pectin, and proteins [36]. Numerous studies have shown that fruit firmness is associated with cell wall polysaccharides such as pectin and cellulose [37,38,39]. The correlation heatmap revealed a positive correlation between the firmness of millet pepper fruits and total dietary fiber, which is consistent with previous findings [40,41]. Vitamin E compounds are antioxidants that protect membrane lipids during biological processes such as photosynthesis and stress responses in plants [42]. Vitamin E also plays a role in reducing the risk of human diseases, with biological functions that include counteracting lipid peroxidation and cooperating with glutathione [43,44]. Seeds are a rich source of vitamin E [45], and since the number of ventricles in chili fruits typically correlates with seed count, this may explain the strong correlation between vitamin E content and the number of ventricles observed in this study. Previous research has shown that capsaicinoids and phenolic compounds are both synthesized via the phenylpropanoid pathway and exhibit antioxidant activity. This shared characteristic underpins the close relationship between capsaicinoid content, total phenolic content, and antioxidant capacity [29], a finding confirmed by our results. Additionally, capsaicinoids are vanillylamides of branched-chain fatty acids [46], and their biosynthesis involves the combined action of fatty acids and phenylpropanoids catalyzed by capsaicin synthase [47]. Our results demonstrated positive correlations between polyunsaturated fatty acids (C20:4n6 and C18:2n6t) and capsaicin, dihydrocapsaicin, and nordihydrocapsaicin, further highlighting the critical role of polyunsaturated fatty acids in capsaicinoid biosynthesis. The correlation analysis also indicated that fruit traits such as flesh thickness and single fruit weight are strongly associated with firmness, springiness, gumminess, and chewiness. This suggests that morphological traits can be used as predictive indicators of fruit texture, facilitating rapid screening for breeding materials or raw ingredients for food processing. Fatty acid composition was found to be closely related to capsaicinoids and vitamin E [48], and the fatty acid profile of different chili varieties has a significant impact on their chemical composition, texture, and color [49]. Thus, fatty acid composition indirectly influences many traits of millet peppers and serves as a key classification criterion. For example, in this study, the germplasms M5, M9, M18, and M24, which exhibited higher fatty acid abundances, clustered into a distinct group.

## 5. Conclusions

This study selected 30 representative millet pepper germplasms to characterize and comprehensively analyze their morphological traits, texture properties, nutrients, fatty acids, and capsaicinoids. The results revealed significant differences among the 30 germplasms in fruit length, fruit width, flesh thickness, stalk length, and single fruit weight, while the variation in the number of ventricles was relatively small. In terms of texture properties, significant differences were observed in firmness, springiness, gumminess, and chewiness, except for cohesiveness. Among these traits, M11 exhibited superior performance compared to other germplasms. The CV results for the nutritional traits indicated that vitamin E, chlorophyll a and b, and total carotenoids showed a wide range of variation among the different germplasms. The Shannon index (H’) results demonstrated that TSS, total sugar, and total carotenoids exhibited higher genetic diversity. Germplasms M5, M18, and M9 contained more than 20 types of fatty acids with high abundances. The capsaicinoid content was highest in M27, M18, and M1, while M11 exhibited the lowest levels, with significant differences observed among these germplasms. The PCA revealed weak correlations between the phenotypic and texture traits and the physicochemical indices, a finding further supported by the correlation heatmap. Additionally, in the PCA analysis based on nutritional traits, capsaicinoids, and fatty acids, the first principal component primarily represented fatty acids. This was consistent with the hierarchical clustering results, where germplasms in PCA group 1 and group 2 corresponded to cluster C1. This study comprehensively characterized and analyzed the differences among these millet pepper germplasms, providing a thorough evaluation of their morphological and nutritional traits. These findings offer valuable data to guide the selection of parental lines in millet pepper breeding programs and provide fundamental data for the development of functional food products.

## Figures and Tables

**Table 1:** Texture parameters of 30 millet pepper germplasms.

	Hardness (N)	Cohesiveness (Ratio)	Springiness (mm)	Gumminess (N)	Chewiness (mj)
M1	8.37 ± 1.59 cde	0.73 ± 0.02 ab	1.35 ± 0.10 bcd	6.27 ± 1.34 cde	8.55 ± 2.35 cd
M2	6.59 ± 0.94 ef	0.76 ± 0.04 a	1.12 ± 0.06 de	5.12 ± 0.65 defg	5.73 ± 0.85 de
M3	11.88 ± 3.47 bc	0.69 ± 0.04 ab	1.30 ± 0.12 bcd	8.41 ± 2.27 bc	11.04 ± 3.62 bc
M4	8.02 ± 2.76 def	0.73 ± 0.06 ab	1.20 ± 0.09 cde	5.96 ± 2.20 cdef	7.25 ± 1.97 de
M5	12.45 ± 2.31 b	0.57 ± 0.10 cd	0.81 ± 0.32 fgh	7.56 ± 2.18 bcd	6.63 ± 1.99 de
M6	7.22 ± 2.13 def	0.54 ± 0.05 cd	0.76 ± 0.09 ghi	4.12 ± 1.26 efg	3.21 ± 1.36 fgh
M7	8.69 ± 3.76 cde	0.53 ± 0.05 cd	0.68 ± 0.12 hi	4.90 ± 2.20 efg	3.46 ± 1.91 fgh
M8	9.28 ± 1.59 bcd	0.56 ± 0.04 cd	0.75 ± 0.12 ghi	5.50 ± 1.09 defg	4.26 ± 1.49 efgh
M9	7.82 ± 1.85 def	0.53 ± 0.06 cd	0.66 ± 0.13 hi	4.52 ± 1.22 efg	3.08 ± 1.37 fgh
M10	5.35 ± 3.29 ef	0.68 ± 0.06 ab	0.82 ± 0.20 fgh	3.76 ± 2.41 fg	3.44 ± 1.14 fgh
M11	18.49 ± 3.43 a	0.58 ± 0.05 cd	1.62 ± 0.10 a	11.28 ± 2.21 a	18.16 ± 3.26 a
M12	10.50 ± 3.57 bcd	0.67 ± 0.03 abc	1.39 ± 0.18 abc	7.40 ± 2.65 bcd	10.33 ± 3.87 bc
M13	7.14 ± 1.65 def	0.71 ± 0.05 ab	0.94 ± 0.09 fg	5.21 ± 1.32 defg	4.90 ± 1.32 efg
M14	9.12 ± 1.98 bcd	0.70 ± 0.03 ab	1.18 ± 0.04 cde	6.62 ± 1.27 bcd	7.85 ± 1.60 de
M15	10.71 ± 3.46 bcd	0.69 ± 0.05 ab	1.48 ± 0.18 ab	7.59 ± 2.15 bcd	11.07 ± 2.66 bc
M16	8.68 ± 1.92 cde	0.74 ± 0.05 ab	1.23 ± 0.17 cd	6.53 ± 1.15 cde	7.97 ± 1.24 de
M17	4.52 ± 0.81 f	0.62 ± 0.05 cd	0.72 ± 0.17 ghi	2.94 ± 0.69 g	2.17 ± 0.79 gh
M18	4.90 ± 1.11 ef	0.65 ± 0.05 bc	0.56 ± 0.11 i	3.29 ± 0.75 fg	1.89 ± 0.74 h
M19	8.21 ± 1.60 cdef	0.69 ± 0.04 ab	1.00 ± 0.14 ef	5.84 ± 1.16 cdef	5.90 ± 1.58 def
M20	7.57 ± 2.88 def	0.70 ± 0.03 ab	1.06 ± 0.18 ef	5.40 ± 2.07 defg	5.90 ± 1.97 def
M21	4.83 ± 1.50 ef	0.66 ± 0.12 bc	0.61 ± 0.06 hi	3.29 ± 1.11 fg	2.03 ± 0.81 gh
M22	7.58 ± 1.59 def	0.67 ± 0.06 abc	0.91 ± 0.07 fg	5.17 ± 0.72 defg	4.74 ± 0.95 efgh
M23	5.75 ± 2.20 ef	0.67 ± 0.04 abc	0.84 ± 0.12 fgh	4.12 ± 1.92 efg	3.55 ± 1.04 efgh
M24	5.65 ± 1.23 ef	0.74 ± 0.04 ab	0.90 ± 0.15 ef	4.28 ± 1.05 efg	3.97 ± 1.53 efgh
M25	7.86 ± 2.00 def	0.65 ± 0.05 bc	0.88 ± 0.13 fg	5.22 ± 0.97 defg	4.59 ± 1.01 efgh
M26	6.85 ± 1.51 def	0.70 ± 0.10 ab	0.99 ± 0.08 ef	4.88 ± 1.07 efg	4.84 ± 1.34 efgh
M27	5.31 ± 1.28 ef	0.76 ± 0.03 a	0.97 ± 0.15 ef	4.14 ± 1.11 efg	4.11 ± 1.47 efgh
M28	5.10 ± 1.17 ef	0.68 ± 0.01 ab	0.90 ± 0.07 fg	3.60 ± 0.83 fg	3.23 ± 0.66 fgh
M29	6.50 ± 1.11 ef	0.70 ± 0.03 ab	1.00 ± 0.09 ef	4.69 ± 0.91 efg	4.70 ± 1.16 efgh
M30	6.84 ± 1.04 ef	0.58 ± 0.08 cd	0.87 ± 0.10 fg	4.18 ± 0.80 efg	3.69 ± 1.06 efgh

Note: The results are shown as the mean ± SD for replicates (for each replicate, n = 10). Different lowercase letters represent significant differences between different germplasms (*p* < 0.05).

**Table 2:** Descriptive statistics for the nutritional quality indicator measurements.

Index	Min	Max	Mean	SD	SE	CV (%)	H’
Chlorophyll a (mg/L)	0.068	0.546	0.234	0.128	0.023	54.724	1.921
Chlorophyll b (mg/L)	0.035	0.408	0.182	0.092	0.017	50.256	1.933
Total carotenoids (mg/L)	0.007	0.377	0.186	0.098	0.018	52.670	1.982
Total soluble solid (%)	3.100	8.730	5.361	1.310	0.239	24.439	1.988
Titratable acid (%)	0.268	0.804	0.478	0.160	0.029	33.486	1.453
Total sugar (mg/g)	6.008	11.900	8.432	1.346	0.246	15.965	1.981
Total phenols (mg/g)	1.363	2.905	1.966	0.425	0.078	21.606	1.907
Vitamin E (μg/g)	13.965	217.334	69.975	66.436	12.129	94.943	1.345
Total dietary fiber (g/100 g)	5.400	15.140	9.078	1.948	0.356	21.459	1.866

Note: SD, standard deviation; SE, standard error; CV, coefficient of variation; H’, Shannon diversity index.

**Table 3:** Fatty acid contents of the 30 millet pepper germplasms (μg/g of sample).

	C4:0	C6:0	C8:0	C10:0	C14:0	C15:0	C15:1	C16:0	C16:1n7	C17:0	C17:1	C18:0	C18:1n9c	C18:2n6t	C18:2n6c	C20:0	C18:3n3	C20:2	C22:0	C22:1n9	C20:4n6	C24:0	SFA	MUFA	PUFA
M1	21.74	13.91	ND	ND	ND	ND	ND	24.15	ND	ND	ND	1.93	7.14	2.41	75.62	ND	20.53	ND	ND	ND	14.49	ND	61.72	7.14	113.05
M2	19.80	13.36	0.02	ND	ND	ND	ND	31.32	1.41	ND	ND	4.66	10.92	0.25	73.20	ND	15.24	ND	ND	ND	6.85	ND	69.16	12.33	95.53
M3	14.84	9.62	0.42	ND	1.41	ND	ND	127.83	5.02	0.55	ND	18.97	68.59	ND	476.66	3.17	22.20	2.57	2.37	1.76	9.64	2.01	181.18	75.37	511.07
M4	14.11	8.82	ND	ND	ND	ND	ND	7.73	ND	ND	ND	0.37	1.81	ND	16.78	ND	8.40	0.34	ND	ND	12.36	ND	31.02	1.81	37.87
M5	13.48	10.04	2.25	2.93	23.60	5.41	13.86	798.46	68.36	10.76	13.87	169.04	466.27	ND	2551.04	19.16	194.07	2.93	20.19	8.90	3.87	21.87	1097.17	571.27	2751.91
M6	23.32	21.67	ND	ND	ND	ND	ND	9.20	ND	ND	ND	18.38	34.37	1.70	ND	ND	9.32	ND	ND	ND	5.13	ND	72.56	34.37	16.15
M7	11.01	8.16	ND	ND	ND	ND	ND	7.87	ND	ND	ND	3.66	8.69	ND	67.75	ND	8.98	0.42	ND	ND	13.55	3.37	34.07	8.69	90.71
M8	25.33	16.26	0.49	ND	ND	ND	ND	15.59	ND	ND	ND	2.94	3.34	ND	22.71	2.47	9.86	0.01	ND	ND	5.20	ND	63.08	3.34	37.78
M9	42.12	24.33	1.88	2.94	22.09	10.94	28.91	427.22	53.31	12.54	17.61	101.91	138.34	ND	898.90	13.60	166.67	2.00	18.48	23.51	6.41	26.29	704.33	261.68	1073.98
M10	7.35	4.14	ND	ND	ND	0.61	ND	2.43	ND	ND	ND	ND	ND	0.35	3.50	ND	10.79	ND	ND	ND	4.62	ND	14.53	0.00	19.25
M11	16.98	12.20	ND	ND	ND	ND	ND	9.12	ND	ND	ND	0.53	3.96	ND	11.02	ND	6.69	0.60	ND	ND	0.70	ND	38.82	3.96	19.01
M12	15.61	8.40	ND	ND	ND	ND	ND	12.31	ND	ND	ND	1.84	3.44	1.27	24.03	ND	15.53	ND	ND	ND	1.16	ND	38.17	3.44	41.98
M13	23.47	15.53	0.53	ND	ND	ND	ND	14.76	ND	ND	ND	2.44	3.59	ND	20.79	2.61	9.63	0.01	ND	ND	5.59	ND	59.33	3.59	36.03
M14	17.79	10.53	0.23	ND	ND	ND	ND	2.02	ND	ND	ND	ND	ND	ND	ND	ND	12.19	ND	ND	ND	2.66	ND	30.56	0.00	14.84
M15	19.93	9.03	ND	ND	ND	ND	ND	13.30	ND	ND	ND	2.57	2.58	ND	42.51	ND	9.78	ND	ND	ND	2.64	ND	44.83	2.58	54.93
M16	24.73	23.54	ND	ND	ND	ND	ND	0.92	ND	ND	ND	ND	ND	ND	ND	ND	0.93	ND	ND	ND	ND	ND	49.19	0.00	0.93
M17	12.89	7.37	ND	ND	5.52	ND	6.49	125.40	28.69	ND	ND	13.39	22.49	0.99	323.86	ND	134.58	ND	ND	ND	ND	ND	164.57	57.67	459.43
M18	10.74	7.90	ND	0.69	6.49	9.40	26.77	320.65	25.71	6.90	7.00	49.88	119.72	ND	809.22	7.56	253.01	1.07	3.93	2.97	19.68	2.66	426.80	182.16	1082.98
M19	7.13	6.11	ND	ND	ND	2.84	ND	3.12	ND	ND	ND	ND	ND	ND	ND	ND	10.59	1.08	ND	ND	5.31	ND	19.20	0.00	16.97
M20	6.03	4.14	ND	ND	ND	0.27	ND	1.64	ND	ND	ND	ND	ND	0.15	1.55	ND	8.26	ND	ND	ND	4.16	ND	12.09	0.00	14.12
M21	11.43	6.88	ND	ND	ND	0.21	0.36	58.18	2.51	0.36	ND	9.32	32.26	ND	140.48	2.12	28.75	0.53	ND	ND	32.42	1.20	89.71	35.13	202.18
M22	16.35	8.29	ND	ND	ND	5.43	ND	1.95	ND	ND	ND	ND	ND	2.06	ND	ND	11.51	ND	ND	ND	11.76	ND	32.02	0.00	25.33
M23	8.22	5.91	ND	ND	ND	2.97	ND	58.04	2.86	0.69	ND	8.62	29.48	0.51	120.14	2.33	20.51	0.57	2.18	1.14	1.76	2.91	91.87	33.48	143.48
M24	8.01	5.83	ND	ND	5.45	1.49	3.17	262.45	22.48	5.21	2.64	42.98	141.93	ND	540.59	6.95	136.59	1.62	4.86	3.84	8.89	2.94	346.18	174.06	687.69
M25	5.81	4.33	ND	ND	ND	0.20	ND	0.32	ND	ND	ND	ND	ND	ND	ND	ND	4.29	ND	ND	ND	0.72	ND	10.66	0.00	5.00
M26	6.58	4.72	ND	ND	ND	0.99	ND	0.90	ND	ND	ND	ND	ND	0.82	ND	ND	10.62	0.35	ND	ND	1.77	ND	13.20	0.00	13.55
M27	5.08	3.33	ND	ND	ND	0.93	ND	6.95	ND	ND	ND	0.35	2.38	1.07	10.42	ND	12.09	ND	ND	ND	1.51	ND	16.64	2.38	25.09
M28	7.96	5.79	ND	ND	ND	ND	ND	0.75	ND	ND	ND	ND	ND	0.43	ND	ND	9.18	0.20	ND	ND	13.15	ND	14.50	0.00	22.96
M29	23.43	13.05	ND	ND	ND	ND	ND	0.16	ND	ND	ND	ND	ND	ND	ND	ND	6.62	1.10	ND	ND	3.44	ND	36.64	0.00	11.16
M30	15.72	8.05	ND	ND	ND	5.72	ND	2.11	ND	ND	ND	ND	ND	2.25	ND	ND	10.46	ND	ND	ND	11.04	ND	31.60	0.00	23.75

Note: ND indicates not detected.

## Data Availability

The data presented in this study are available from the corresponding authors upon reasonable request.

## Supplementary Materials

The following supporting information can be downloaded at: https://www.mdpi.com/article/10.3390/metabo15010047/s1, Table S1: Morphological indicator measurements of fruit for the 30 millet pepper germplasms, Table S2: Nutritional indicators measurements of fruit for the 30 millet pepper germplasms, Table S3: Capsaicinoid contents, Table S4: Correlation analysis results of all indicators.
